# Lichtenstein versus transabdominal preperitoneal (TAPP) inguinal hernia repair for unilateral non recurrent hernia: A multicenter short term randomized comparative study of clinical outcomes

**DOI:** 10.1016/j.amsu.2022.103428

**Published:** 2022-03-18

**Authors:** Ahmed Abd El Aal Sultan, Hossam Attia Abo Elazm, Hisham Omran

**Affiliations:** a General Surgery, Faculty of Medicine – Al-Azhar University, Egypt; b General Surgery, Faculty of Medicine – Ain Shams University, Egypt

**Keywords:** Lichtenstein repair, Trans-abdominal pre-peritoneal repair, Inguinal hernia, TAPP, Post-operative pain

## Abstract

**Background:**

The repair of inguinal hernia is still one of the most prevalent surgical procedures done worldwide. Among all repair techniques, open Lichtenstein repair is the most globally conducted. In the past few decades, laparoscopic technique for inguinal hernia repair has increased technical demand. *Trans*-abdominal pre-peritoneal (TAPP) technique is the main approach, which is featured by less postoperative pain and early recovery.

**Objective:**

The current work is a short-term study to make a comparison between open Lichtenstein repair of inguinal hernia as well as laparoscopic *trans*-abdominal preperitoneal repair of inguinal hernia (TAPP) for unilateral non recurrent hernia regarding intraoperative, postoperative complications and hospital stay.

**Patients and methods:**

The present prospective randomized study recruited 100 male subjects from General Surgery Department of Al-Azhar University Hospitals and Ain Shams university hospitals suffering from oblique inguinal hernia (unilateral non recurrent hernia) with an age above 18 years and good overall health, who were randomized into two groups: Group A: 49 cases were subjected to laparoscopic *trans*-abdominal pre-peritoneal repair (TAPP). Group B: 51 cases were subjected to open Lichtenstein repair.

**Results:**

This study detected less post-operative pain day 0, day 1, day 7 and 1 month postoperatively. There was no significant difference at 6 months post-operatively.

**Conclusion:**

Finally, we concluded that TAPP repair for inguinal hernia (unilateral non recurrent hernia) safer with less early post-operative pain. Also, it has fewer complications, with a significantly longer operative time.

## Highlights

• Inguinal hernia can be treated with either open surgical technique (Lichtenstein) or laparoscopic technique (transabdominal preperitoneal repair).
• TAPP repair for inguinal hernia safer with less early post-operative pain.
• There is no statistical difference as regard to surgical complications and post-operative recurrence.

## 1 Introduction

***Courtney et al.*** [1] defined hernia as an odd cusp of part of the abdominal cavity contents in the form of a disorder in its surrounding walls and repair of hernia is a prevalent operation conducted by general surgeons. In spite of this procedure frequency, no optimal results are obtained; as it is correlated with some complications like nerve injury, postoperative pain, infection, as well as recurrence remain.

***McCormack et al.*** [2] mentioned that the standardized inguinal hernia repair method has been slightly modified over the past years until introducing synthetic mesh. The mesh can be placed by utilizing the open approach or performing a minimally invasive laparoscopic access approach. There are no substantial differences in recurrence incidence between laparoscopic as well as open mesh hernia repair techniques. The study detected less pain and numbness after laparoscopic repair, In addition to faster returning to everyday activities.

Nevertheless, operation time is much higher along with elevated serious vascular injury risks**.**

***John and Andrew*** [3] stated that regardless of this disease pervasiveness, there is not any globally agreed upon classification system. Consequently, a wide variety of cases experience inguinal hernias. Due to this controversy, there is no unified technique for repair that has the potential to manage all inguinal hernia cases. Hence, surgeons who perform inguinal hernias have to identify both of laparoscopic as well as open procedures in order to grant patients the optimum method of repair based on distinguished factors of patients as well as characteristics of the hernia defect**.**

***Olmi et al.*** [4] reported that techniques of laparoscopy have been increasingly utilized in the repair of inguinal hernias offering the potential advantages of minimally invasive surgery, possibly a lower recurrence rate and lower cost according to a randomized controlled study.

Preperitoneal placement of the mesh during unilateral inguinal hernia repair shows excellent outcomes, regardless of the surgical approach used [5].

***Hwang et al.*** [6] found that laparoscopic repair has proven to be efficient in several recurrent or primarily inguinal hernia cases as well as scores in low recurrence rates, along with elevated scores of patient satisfaction**.**

## 2 AIM of the work

The present study aims to make a comparison between open Lichtenstein repair of inguinal hernia as well as laparoscopic *trans*-abdominal preperitoneal repair of inguinal hernia (TAPP) for unilateral non recurrent hernia regarding intraoperative, postoperative complications and hospital stay.

### 2.1 Patients and Methods

**The present prospective randomized study recruited 100 male subjects who were categorized into two groups; *Group A:*** 49 cases underwent laparoscopic transabdominal pre-peritoneal repair (TAPP). ***Group B:*** 51 patients underwent open Lichtenstein repair. Ethical approval from ethical committee was obtained, as well as a written consent from patients.

**Inclusion criteria:** Patients involved in this study included those attended the General Surgery Department of General Surgery Al-Azhar University Hospitals and Ain Shams university hospitals suffering from oblique inguinal hernia for unilateral non recurrent hernia with an age above 18 years and good overall health, in period from January 2020 to January 2021.

**Exclusion criteria:** Female sex as well as cardiac, hepatic, uremic, and uncompensated pulmonary disease patients. In addition to, complicated group, previous abdominal operations, bilateral inguinal hernia, as well as recurrent group.

**All patients included in the study were subjected to:** History taking, general examination and routine preoperative investigations. Besides obtaining informed written consent. Evaluations of the operative time, blood loss, mesh size and material, method, material of mesh fixation, and any intra-operative complications. Postoperative evaluation of pain score (NRS), need for analgesia, hospital stay duration as well as post-operative complications. A six-month follow-up was performed in order to compare the efficacy as well as satisfaction of patients in both groups. the work has been reported in line with the STROCSS 2021 criteria [7].

**Anesthesia:** General anesthesia for laparoscopic cases. Spinal anesthesia for open cases.

#### 2.1.1 Operative technique

**In laparoscopic repair** we used **TAPP** technique**:** fixation of the mesh was done by using tacker, closure of peritoneum by staple or suture**.**

**In open repair,** we used **lichtenstein** technique**.**

#### 2.1.2 Post-operative

After recovery, the patient will be sent to the inpatient ward. Feeding will start 6 h post-operatively with a prescription of paracetamol (IV) whenever needed as an analgesic.

The patient will be discharged next day postoperative with follow up after one week for assessment of short-term complications including pain score (NRS), use of analgesia, scrotal edema and resumption to usual activity.

6 months post –operatively, patients will be asked for coming back for follow up and recording long term complications patient satisfaction (which is 0–10 analogue scale).

#### 2.1.3 Statistical analysis

The data was tabulated as well as processed via SPSS (26) statistical package for Windows 7:

Quantitative variables were expressed by means as well as standard deviation, and then the analysis was performed utilizing the independent sample *t*-test.

Qualitative data was expressed in the form of frequency as well as percentages, and then the analysis was done via Chi square test, but when more than 20% of cells have expected frequencies <5, and the test of Fisher's exact was utilized.

The findings were significant when p value < 0.05 and highly significant when p value < 0.01.

## 3 Results

The results can be summarized in the following tables: (see Table (1), Table (2), Table (3), Table (4)).

**Table (1):** Age in both groups.

		TAPP (49)	Lichtenstein (51)	Test value	P value	significance
**Age**	mean ± SD)	34.71 ± 11.95	35.82 ± 11.45	0.474	0.673	NS
	Range	21–65	24–66			

independent sample *t*-test.

**Table (2):** Operative time.

		TAPP (49)	Lichtenstein (51)	Test value	P value	significance
**Operative time (min)**	mean ± SD	93.78 ± 17.24	72.39 ± 18.21	6.026	0.000	HS
	range	60–130	45–110			

independent sample *t*-test.

**Table (3):** Comparison of post-operative pain scores.

		TAPP (49)	Lichtenstein (51)	Test value	P value	significance
**Day 0**	mean ± SD	1.80 ± 0.87	3.29 ± 0.92	8.366	0.000	HS
	range	0–4	1–5			
**Day 1**	mean ± SD	1.22 ± 0.80	2.41 ± 0.98	6.615	0.000	HS
	range	0–2	1–4			
**1 Week**	mean ± SD	0.16 ± 0.37	0.80 ± 0.78	5.297	0.000	HS
	range	0–1	0–2			
**1 Month**	mean ± SD	0000	0.12 ± 0.33	2.582	0.013	S
	range	0–0	0–1			
**6 Months**	mean ± SD	0000	0000	NA	NA	NA
	range	0–0	0–0			

independent sample *t*-test.

No Intra operative complications in both groups.

**Figure:**
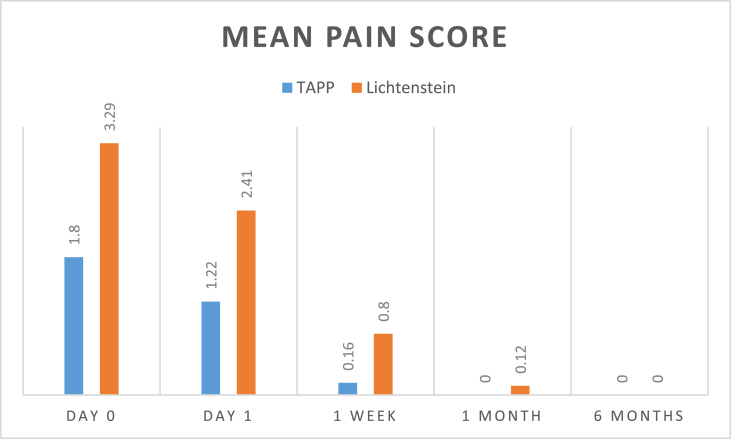

**Figure:**
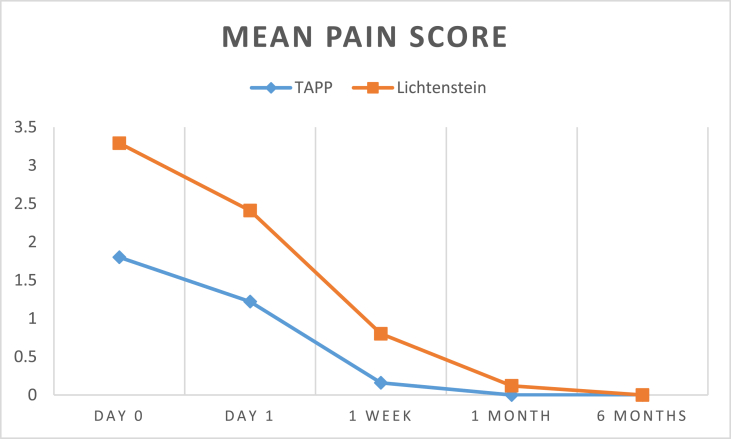

**Figure:**
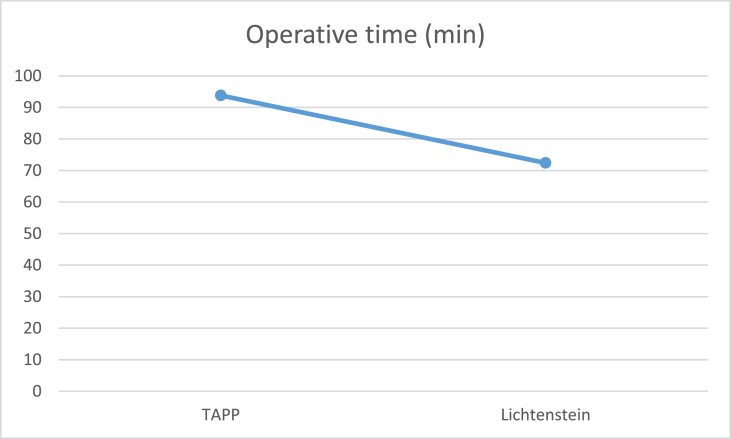

**Figure:**
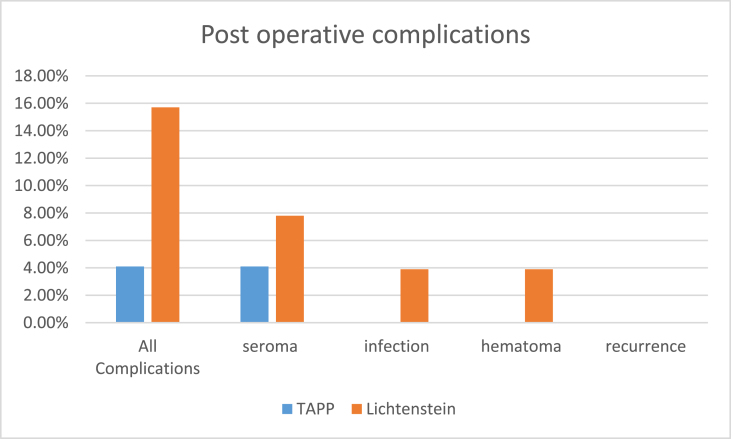

**Table (4):** Comparison of post-operative complications.

		TAPP (49)	Lichtenstein (51)	Test value	P value	significance
**Overall Complications**	yes	2(4.1%)	8(15.7%)	1.924^a^	0.092	NS
	no	47(95.9%)	43(48.3%)			
**Seroma**	Yes	2(4.1%)	4(7.8%)	0.788^a^	0.678	NS
	No	47(95.9%)	47(92.2%)			
**Infection**	Yes	0(0%)	2(3.9%)	1.393^a^	0.495	NS
	No	49(100%)	49(96.1%)			
**Hematoma**	Yes	0(0%)	2(3.9%)	1.393^a^	0.495	NS
	No	49(100%)	49(96.1%)			
**recurrence**	Yes	00	00	NA	NA	NA
	No	49(100%)	51(100%)			

a Fisher's exact test.

## 4 Discussion

***Antoniou et al.*** [8] stated that the repair of inguinal hernia is one of the most globally performed surgical procedures.

Lichtenstein repair is the most common technique done for repair. Nonetheless, in the last years laparoscopic technique for repair of inguinal hernia is technically demanded mainly as (TAPP) technique.

***Claus et al.*** [9] mentioned that TAPP approach necessitates the minimally invasive surgery benefits, like pain relieve as well as early recovery.

### 4.1 Operative time

***Simons et al.*** [10] demonstrated that the median operative time was moderately elevated in the TAPP as compared to the open Lichtenstein repair technique (110.3 vs. 97.1 min; p = 0.23).

In laparoscopic TAPP repair, the use of partially absorbable mesh is more better than the use of nonabsorbable mesh regarding postoperative pain and time needed to return to routine daily activities, but was accompanied with longer operative time [11].

In the present study, the mean operative time was (93.78 ± 17.24) minutes for TAPP, (72.39 ± 18.21) minute for open Lichtenstein repair. It can be attributed to the small sample size in this study.

### 4.2 Intra-operative complications

***Neumayer et al.*** [12] illustrated that intra-operative complications were more in a laparoscopic procedure.

Indeed, the skills of surgeons in laparoscopic repair make a difference. Also, spermatic cord structures demonstrated less that injured in TAPP compared to the open method, possibly due to the laparoscopic view which is magnified.

In this study, none of the recruited subjects experienced intra-operative complications. This can be attributed to the small sample size in our study.

### 4.3 Post-operative complications

Grant [13] displayed substantially more diminished wound infection occurrence as well as hematoma along with elevated occurrence of seromas following laparoscopic repair.

In our study, there was no patients with wound infection (0%), two patients with seroma (4.1%), no patients with hematoma (0%), for TAPP repair in contrast to 4 seroma cases (7.8%), two patients with hematoma (3.9%) and two patients with wound infection (3.9%); however, no marked differences were detected between both groups.

### 4.4 Hernia recurrence

**Schmedt *et al.*** [14] found a more elevated recurrence rate after the endoscopic repair.

In our study, there were no substantial differences in terms of hernia recurrence, which may be due to the short period of follow up and the small number of patients.

### 4.5 Post-operative pain

***Wennergren et al.*** [15] stated that laparoscopic inguinal hernial repair is correlated with more releiving early post-operative pain in contrast to the open Lichtenstein repair.

***Wijerathne et al.*** [16] clarified that complications as well as postoperative pain are significantly correlated. In addition, less post-operative pain may be attributed to fewer complications, which are associated with this approach.

TAPP repair was associated with earlier toleration of oral feeds, lesser post-operative pain, earlier discharge from the hospital, earlier return to usual activities, and less persisting pain [17].

Our study detected less postoperative pain day 0, day 1 as well as post-operative day 7 in TAPP repair with a highly significant P value. There was a significant difference 1 month post-operatively. No significant differences were detected 6 months post-operatively.

One limitation to this work is the relatively small sample size. Another limitation is the relatively short follow up period of 6 months with the possibility of missing long-term cumulative benefit of the surgery.

## Conclusion

Our study showed that TAPP repair of inguinal hernia is safer with less early post-operative pain. Also, it has fewer complications, with a significantly longer operative time.

## Provenance and peer review

Not commissioned, externally peer reviewed.

## Ethical approval

Approval from ethical committee & written consent from Pt. was obtained.

## Sources of funding

Self-funding

## Author contribution

All authors contributed in equal manner.

## Registration of research studies

1. Name of the registry: Researchregistry
2. Unique Identifying number or registration ID: researchregistry 7487
3. Hyperlink to your specific registration (must be publicly accessible and will be checked): http://www.researchregistry.com/browse-registry#home/

## Guarantor

Ahmed Abd El Aal Sultan, E mail: dr.ahmedsultan2azhar.edu.eg, ORC ID: 0000-0003-1097-2615.

## Declaration of competing interest

No conflict of interest.
